# Evaluation of three commercial bovine ELISA kits for detection of antibodies against Alphaherpesviruses in reindeer (*Rangifer tarandus tarandus*)

**DOI:** 10.1186/1751-0147-51-9

**Published:** 2009-03-09

**Authors:** Carlos G Das Neves, Matthieu Roger, Nigel G Yoccoz, Espen Rimstad, Morten Tryland

**Affiliations:** 1 The Norwegian School of Veterinary Science, Department of Food Safety and Infection Biology, Section of Arctic Veterinary Medicine, Stakkevollveien 23, NO-9010 Tromsø, Norway; 2 University of Tromsø, Institute of Biology, NO-9037 Tromsø, Norway; 3 The Norwegian School of Veterinary Science, Department of Food Safety and Infection Biology, Section of Microbiology, Immunology and Parasitology, PO Box 8146, NO-0033 Oslo, Norway; 4 YROI: Cyclotron et Recherche Biomédicale Technopole – 2 Rue Maxime Rivière – BP 80005 – 97491 Sainte Clotilde Cedex, Island of Réunion

## Abstract

**Background:**

The genus *Varicellovirus *(family *Herpesviridae *subfamily *Alphaherpesvirinae*) includes a group of viruses genetically and antigenically related to bovine herpesvirus 1 (BoHV-1) among which cervid herpesvirus 2 (CvHV-2) can be of importance in reindeer. These viruses are known to be responsible for different diseases in both wild and domestic animals. Reindeer are a keystone in the indigenous Saami culture and previous studies have reported the presence of antibodies against alphaherpesviruses in semi-domesticated reindeer in northern Norway. Mortality rates, especially in calves, can be very high in some herds and the abortion potential of alphaherpesvirus in reindeer, unlike in bovines, remains unknown.

ELISA kits are the most used screening method in domestic ruminants and given the close genetic relationship between viruses within this genus, it might be possible to use such kits to screen cervids for different alphaherpesviruses. We have compared three different commercial ELISA kits in order to validate its use for reindeer and CvHV-2.

**Methods:**

Three commercial bovine ELISA kits (A, B and C), using either indirect (A) or blocking (B and C) ELISA techniques to detect antibodies against BoHV-1 were tested with sera from 154 reindeer in order to detect antibodies against CvHV-2. A Spearman's rank-based coefficient of correlation (ρ) was calculated. A dilution trial was performed for all kits. A virus neutralization test using both BoHV-1 and CvHV-2 was carried out.

**Results:**

Seroprevalence was almost the same with all kits (40–41%). Despite a similar qualitative score, quantitatively kits classified samples differently and a strong correlation was only identified between Kits B and C. Blocking kits performed better in both repeatability and in the dilution trial. The virus neutralization results confirmed the ELISA results to a very high degree. Neutralizing titres ranged from 1:2 to 1:256 and from 0 to 1:16 against CvHV-2 and BoHV-1 respectively.

**Conclusion:**

Results show that the genetic and antigenic similarity between BoHV-1 and CvHV-2 enables the use of a bovine gB blocking ELISA kit to screen reindeer. The use of an ELISA kit is both cheaper and time saving, allowing screening of large populations. This study revealed a high number of positive animals against CvHV-2 and its impact and distribution in the general population should be further evaluated.

## Background

Viruses in the genus *Varicellovirus *(family *Herpesviridae *subfamily *Alphaherpesvirinae*) are known to infect and cause disease in several ruminant species. Of the alphaherpesviruses infecting ruminants bovine herpesvirus type 1 (BoHV-1), causing the diseases Infectious Bovine Rhinotracheitis (IBR) and Infectious Pustular Vulvovaginitis (IPV), is well-described [1,2]. Other viruses of this genus related to BoHV-1 are known to cross-react serologically and have been isolated from semi-domesticated and wildlife ruminant species such as cervid herpesvirus 2 (CvHV-2, also known as Rangiferine Herpesvirus, RanHV) from semi-domesticated reindeer (*Rangifer tarandus tarandus*) in Finland and Sweden [3,4]. Serological evidence of alphaherpesvirus infection in reindeer has further been reported in Greenland [5] and Alaska [6] as well as in both wild [7] and semi-domesticated reindeer [8-10] in Norway, although it is unknown which alphaherpesvirus is circulating in these populations.

Finnmark County in northern Norway (55 047 km^2^) is the largest reindeer herding area in Norway with an estimate of 168 779 animals in 2005/2006 [11]. In this area the reindeer are kept in a semi-nomadic way being herded between summer and winter pastures, and being usually free-ranging within the borders of their specific herding districts. Mortality rates in reindeer in Finnmark vary significantly between years and reached 47% for calves in 2005–2006 [11]. The impact of CvHV-2 in reindeer mortality or abortion, events commonly associated with other alphaherpesvirus infections in ruminants [12], remains unknown.

In Norway the last BoHV-1 infection in cattle was reported in 1993 [13], and the country has officially eradicated IBR/IPV although a surveillance program is still ongoing. According to previous serosurveys [9,10], alphaherpesvirus infections are suspected in semi-domesticated reindeer in Finnmark, which is of great epidemiological importance since cross-species infections between bovines and reindeer have been shown for BoHV-1 and CvHV-2 [12].

Many countries use sero-epidemiological surveys of bovine populations to maintain an active surveillance or to eradicate IBR/IPV. Different methods for screening for antibodies against BoHV-1 in cattle have been developed in several countries. In a study comparing serological BoHV-1 tests, a blocking Enzyme Linked Immunosorbent Assay (ELISA) based on glycoprotein B (gB) antigen was found to be the best option with a sensitivity of 96% and a specificity of 99% [14]. This was a better score than other blocking ELISAs based on other glycoprotein antigens (glycoprotein E), indirect ELISAs or virus neutralization tests (VNT) [14].

Glycoprotein B plays a decisive role in the interaction between the virus and host cells during the attachment, penetration and replication processes of the virus [12]. The nucleotide sequence encoding gB is highly conserved between BoHV-1 and CvHV-2 [15,16].

Serological cross-reactions have been shown to exist between different viruses within the *Varicellovirus *genus and several studies have calculated coefficients of antigenic similarity (R) proving the serological cross-reactivity between CvHV-2 and BoHV-1 [17-20].

Given the serological cross-reactions within this genus, serological tests for BoHV-1 based on highly conserved antigen, such as gB, could be used to detect the presence of antibodies against alphaherpesviruses in non-bovine ruminant host species. Since these viruses generally establish latency and life-long infections in their natural hosts, the presence of antibodies most likely indicates that the animals are persistently infected.

There are no standardized methods to conduct serological testing of reindeer populations and different serological techniques have been used in smaller sero-surveys carried out in Alaska [6,21], Norway [7-10] and Greenland [5]. Simultaneously, IBR/IPV eradication campaigns have many times neglected the status of wild animals as possible reservoir species for alphaherpesviruses.

To assess the present alphaherpesvirus infection status of reindeer from different reindeer husbandry districts in Finnmark, a reliable and feasible serological test was needed. Three commercial ELISA kits for detecting antibodies against BoHV-1 in cattle were evaluated regarding their ability to detect antibodies against alphaherpesviruses in reindeer: one indirect ELISA with BoHV-1 as antigen, and two blocking ELISA kits with BoHV-1 gB as antigen.

## Methods

### Origin of samples

A total of 154 serum or plasma samples from four geographically separated herds from Finnmark County, representing adults and calves as well as both genders, were collected in 2004–2005.

### Serological testing

The samples were analyzed in duplicate in all the three commercial kits. The main characteristics for these kits (A, B and C) are presented in Table 1. The manufacturer's instructions and kit components were used in Kits B and C, while for Kit A adaptations were necessary.

**Table 1:** Major characteristics and modifications of the three commercial bovine ELISA kits used to test reindeer for alphaherpesvirus antibodies in this study.

	**ELISA type**	**Well antigen**	**2nd antibody**	**Revelation system**	**Absorbance**	**Validation rules**
**SVANOVA – A**	Indirect	BoHV-1 unknown antigen in one well and cells on another.	*Rabbit anti-reindeer antibody*	*Streptavidin-POD + OPD*	*450 nm*	*OD*_*S *_= (OD_*IBR *_- OD_*CONTROL*_)*Sample is positive if OD_*SAMPLE*_>0*
**SYNBIOTICS – B**	Blocking	BoHV-1 gB antigen	2 monoclonal antibodies (Mabs) anti-gB/peroxidase	Peroxidase system	450 nm + 620 nm (for correction)	Validation rules: %P = [(OD_N _- OD_P_)/OD_N_] ×100 > 80% and OD_N_>0,500 Sample is positive if: %S = [(OD_N _- OD_S_)/(OD_N _- OD_P_)] ×100 > 60 Sample is doubtful if: 45<%S<60
**LSI – C**	Blocking	BoHV-1 gB antigen	1 monoclonal antibody anti-gB/HRP labelled	Horseradish Peroxidase	450 nm + 620 nm (for correction)	Validation rules: %P = [(OD_N _- OD_P_)/OD_N_] ×100 > 70% and OD_N_>0,700 Sample is positive if: %S = [(OD_N _- OD_S_)/(OD_N _- OD_P_)] ×100 > 50 Sample is doubtful if: 45<%S<50

**BoHV-1**: Bovine herpesvirus type 1

**gB**: Glycoprotein B

**OPD**: orthophenyldiamine

**OD**_**N**_: Mean optical density of the negative control sera

**OD**_**P**_: Mean optical density of the positive control sera

**OD**_**S**_: Mean optical density of the sample sera

*Changes of the protocol to adapt the kit to reindeer are depicted in italic*.

Kit A, Infectious Bovine Rhinotracheitis (IBR-Ab) SVANOVIR™ (Svanova Biotech AB Sweden), is an indirect ELISA. The test wells are coated with a mixture of viral and cellular proteins from virus-infected cells whereas control wells are coated with material from non-infected cells of identical type. The test serum samples were diluted 1:25 and added to test and control wells. Kit A is based on an indirect method, and because of this the secondary antibodies provided with the kit (horseradish peroxidase conjugated anti-bovine IgG monoclonal antibodies) could not be used, as they would not recognize reindeer antibodies. They were therefore replaced by a biotin labeled rabbit-anti-reindeer antibody in a 1:200 dilution and incubated for 1 h at 37°C [22]. Revelation was achieved using Streptavidin-β peroxidase (POD-conjugate) diluted 1:10000 (Roche^® ^Mannheim, Germany) and incubated for 1 h at 37°C, followed by orthophenyldiamine (OPD) as substrate (DakoCytomation^® ^Glostrup, Denmark) incubated for 10 min in the dark at 20°C. The enzyme reaction was stopped by adding 100μL of 1 M H_2_SO_4 _per well.

Because positive and negative controls of the ELISA kit were from cattle, they could not be used in an indirect ELISA method where the secondary antibody was replaced. The validation criteria proposed by the manufacturer could hence not be used, and samples were therefore considered positive when the mean OD of the antigen well minus the mean OD of the control well was above zero, which indicates a higher reaction in the antigen well compared to the control well.

Kit B, SERELISA™ IBR/IPV gB Ab Mono Blocking (SYNBIOTICS EUROPE SAS, France) is a blocking ELISA in which two peroxidase conjugated monoclonal antibodies against the gB protein of BoHV-1 compete with the serum sample antibodies in binding to gB antigens in the well. The negative and positive control sera from cattle supplied with the kit were used. The test serum samples were diluted 1:2. A competition percentage was calculated based on the relation between the OD mean of the duplicates and of the controls. Samples with a competition percentage above 60% were considered seropositive, between 45–60% doubtful and below 45% seronegative, as recommended when testing cattle serum samples.

Kit C, gB BLOCKING LSI™ (LSI, France – Laboratoire Service International), is based on the same blocking design as Kit B, but with one monoclonal antibody against the gB protein of BoHV-1 labeled with horseradish peroxidase (HRP). The negative and positive control sera from cattle supplied with the kit were used and test serum samples were diluted 1:2. A competition percentage was calculated as for Kit B. Samples with a competition percentage above 50% were considered seropositive, between 45–50% doubtful and below 45% seronegative, as recommended for cattle.

### Sample dilution curves

In order to verify the analytical sensitivity of these kits, a serial dilution of a panel of four selected serum samples was performed in parallel for each kit. The starting point was the initial serum dilution used for each kit (1:25 in Kit A and 1:2 in Kits B and C). A twofold dilution was conducted, in Kit A from 1:25 to 1:3200, and in Kits B and C from 1:2 to 1:256. The four samples chosen were all from herd IV: serum sample 24 was strongly positive in all kits; serum sample FA16 was moderately positive in all kits; serum sample FA15 was classified as doubtful in Kit B and seronegative in Kits A and C, and serum sample FB15 was negative in all kits.

For Kits B and C the respective positive and negative control sera from cattle were also tested. For Kit A an additional sample of water was added as a negative control and diluted as the other samples using the kit's dilution buffers.

All dilutions were tested in duplicate and mean optical density (OD) values were obtained according to the kit's specifications and used for calculations.

### Virus neutralization test (VNT)

Given the serological cross-reaction between BoHV-1 and CvHV-2 and considering that the ELISA kits were designed for cattle, VNT was performed on all the reindeer serum samples to further validate the use of these kits in reindeer and to confirm their ability to detect antibodies against CvHV-2.

Reindeer sera were two fold diluted and each dilution (from 1:2 to 1:256) was incubated with 100 TCID_50 _of CvHV-2 or BoHV-1 at 37°C for 1 h.

A mixture of serum and virus (50 μl) was added to wells in 96 well plates. To each well, 100 μl of Madin-Darby bovine kidney cells (MDBK), with calculated area coverage of 100%, was added. The medium used was Earles MEM with addition of 2% foetal calf serum (FCS) and 2% penicillin-streptomycin (PS 10 000 Units/mL penicillin and 10 mg/mL streptomycin, SIGMA-ALDRICH, Oslo Norway). The plates were incubated for 2 days and then stained according to manufacturer's protocol (Diff-Quik Staining Protocol, Hamilton Thorne Research). Reading was performed and titres expressed as the reciprocal of the highest serum dilution that completely prevented a cytopathic effect (CPE). A reindeer serum sample, obtained from an animal experimentally infected with CvHV-2, and a bovine serum sample, obtained from a bovine infected with BoHV-1, were added as positive controls and used to calculate the coefficient of antigenic similarity (R) as previously described by Lyaku et al. [18].

### Statistical analysis

As all samples were tested in duplicates, repeatability was assessed using the absolute difference between the OD values (variability) calculated for each sample in each kit.

As the distribution of absolute difference was highly skewed, the 5–95% quantiles (i.e. an interval including 90% of observations with 5% on either side) were used instead of standard deviation to describe distribution of individual values.

Because using ranks resulted in more robust statistics [23], we used Spearman correlation (ρ) to assess the relationships between kits. Calculations were done for two sub-samples: observations below and above the cut off lines to assess the relationships between the different kits for the populations of negative versus positive samples in general and around the cut-off values. The squared value ρ^2 ^can be interpreted in terms of predictive power (explained variability) of one kit's ranks by the other kit's ranks. P-value was considered significant if below 0.05. All calculations were done in R (R Development Core Team 2008).

## Results

### Serological testing

**Figure 1:**
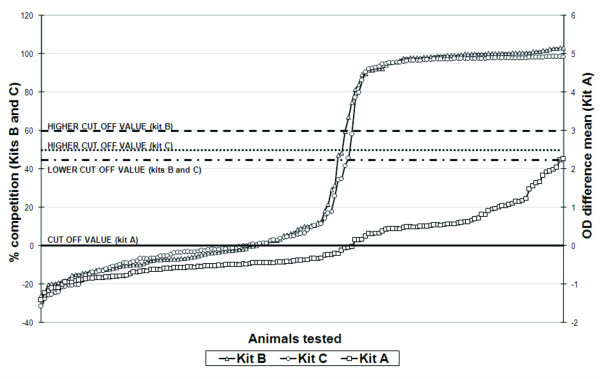
**Serology results**. Serology results for 154 samples of semi-domesticated reindeer from Finnmark County, Norway displayed in ascending OD for Kit A and in ascending competition percentage for Kits B and C.

The three ELISA kits produced very similar seroprevalences results. Kit A classified 62 reindeer as having antibodies against alphaherpesvirus (40.3%); Kit B 64 seropositive reindeer (41.6%) and three classified as doubtful and Kit C 63 seropositive reindeer (41.0%) with one animal classified as doubtful. Results were arranged in ascending order according to OD difference mean values for Kit A and to competition percentages for Kits B and C (Figure 1). The curves confirm that the individual results were distributed following a sigmoid curve for all three kits, although a more flattened curve was produced by Kit A. For Kits B and C most individuals were clustered in two distinct groups, one up to 20% of competition, representing the negative samples, and the other from 85% upwards, representing the individuals classified as seropositive.

Both positive and negative controls for Kit B and C scored well above the manufacturer's required thresholds.

To reveal if different kits were presenting similar qualitative results (positive, negative or doubtful) a scatter plot, displaying the results for each animal in each kit compared two by two, was constructed (not displayed). Comparing Kits B and C three animals were classified as doubtful in Kit B and seronegative in Kit C, and one animal was seropositive in Kit B and classified as doubtful in Kit C. Comparing Kit A and B two animals were classified seronegative in Kit A and seropositive in Kit B, whereas three animals were classified negative in Kit A and doubtful in Kit B. Comparing Kit A and C one animal was classified seronegative in Kit A and doubtful in Kit C.

**Figure 2:**
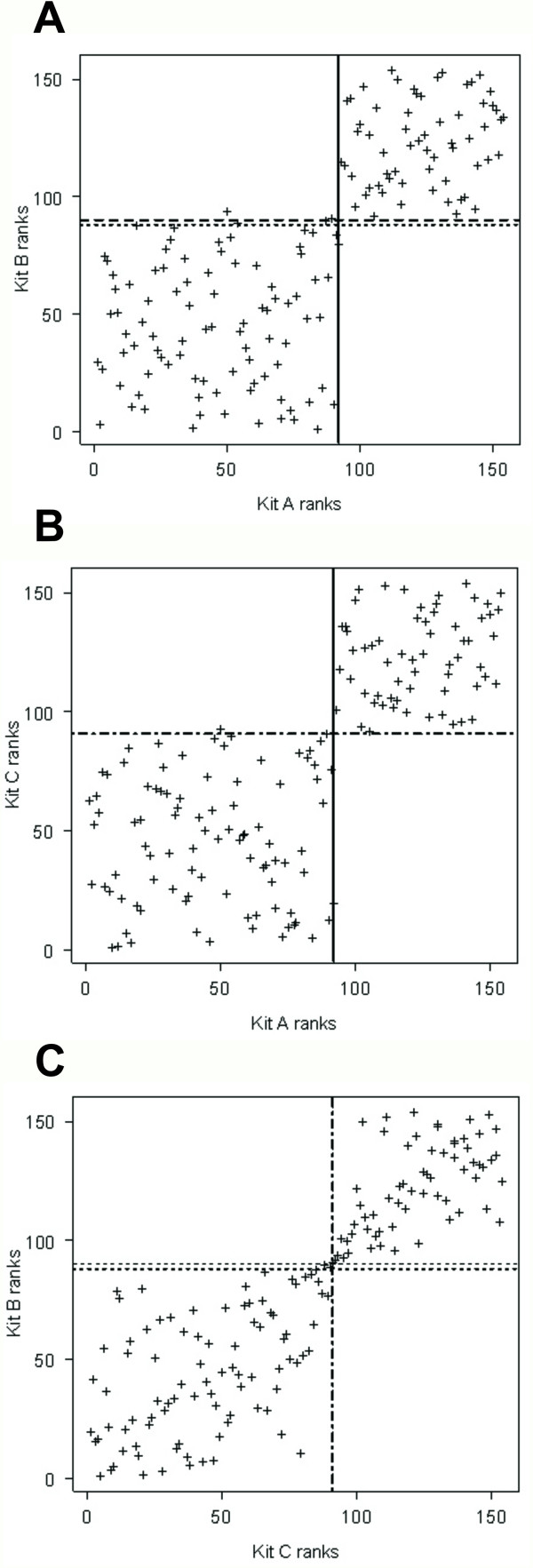
**Comparison of three serological kits for detecting antibodies against alphaherpesvirus in reindeer by the ranks of the results**. The comparison of the kits two by two was done by plotting ranks after sorting the results (OD values for Kit A and competition percentage for Kit B and C) in ascending order. Results were given a rank position: 1^st ^rank being the most negative and 154^th ^rank the most positive. The graphs display the rank obtained per animal in each kit. Lines pass through the rank closest to the cut-off values for each kit (Kit A cut off value 0; Kits B and C lower cut off value 45%; Kit B higher cut off value 60%; Kit C higher cut off value 50%). For Kit A, a line (—) passes through the 92^nd ^rank (-0.031). For Kit B, a line (···) passes through the 88^th ^rank (47.28%) and another (— —) passes through the 90^th ^rank (59.60%). For Kit C, a line (— – —) passes through the 91^st ^rank (45.54%) and represents both cut-off values (higher and lower) as no samples were ranked in between. 2A: scatter plot displays Kit A and Kit B correlation. 2B: scatter plot displays Kit A and Kit C correlation. 2C: scatter plot displays Kit B and Kit C correlation.

Spearman coefficients (variability of one kit's ranks explained by the other kit's ranks) showed that, despite an almost absolute agreement of qualitative results (samples being classified as positive, negative or doubtful) between the kits, the quantitative results were not as concurrent (Figure 2 and Table 2). In fact, only between Kits B and C (Figure 2C) was there evidence for a strong correlation in ranks both for negative as well as positive samples (P < 0.001). A restricted analysis of samples in the slope of the curve, shown in Figure 1 (approximately ranks between 68^th ^and 111^th ^in Figure 2), confirmed the general observations. A correlation was evident between Kits B and C for which two clear sub-populations, negative and positive, outflanking the cut-off value were identified and confirmed by VNT. No evidence for a correlation was found within seropositive or seronegative animals by other kit comparisons (Figure 2A and 2B) apart from a weak positive association within positive results for Kit A and Kit B (Figure 2A), (P = 0.049; all other P-values > 0.09, Table 2).

**Table 2:** Spearman correlation analysis within positive and negative results for the three commercial bovine ELISA kits tested, compared two by two.

**Kits compared**	**Population analysed**	**ρ**	**P**^1^
*Kit A and B*	Negative (<88^th ^rank for Kit B and <92^nd ^rank for Kit A)	0.032	0.767
	Positive (>90^st ^rank for Kit B and >92^nd ^rank for Kit A)	0.247	0.049

*Kit A and C*	Negative (<91^st ^rank for Kit C and <92^nd ^rank for Kit A)	-0.003	0.974
	Positive (>91^st ^rank for Kit C and >92^nd ^rank for Kit A)	0.213	0.093

*Kit B and C*	Negative (<88^th ^rank for Kit B <91^st ^rank for Kit C)	0.481	**<0.001**
	Positive (>90^th ^rank for Kit B and >91^st ^rank for Kit C)	0.593	**<0.001**

^1 ^P-value is considered significant if below 0.05

### Repeatability analysis

Kit A had the highest variability between OD duplicates with a maximum difference in optical density of 2.35 and a mean difference of 0.37 (5–95% quantiles: [0.018; 1.188]). Kit B had a maximum difference of 0.30 and a mean of 0.03 (5–95% quantiles [0.001; 0.160]) and Kit C a maximum difference of 0.27 and mean of 0.06 (5–95% quantiles [0.001; 0.123]).

### Serial dilution results

**Figure 3:**
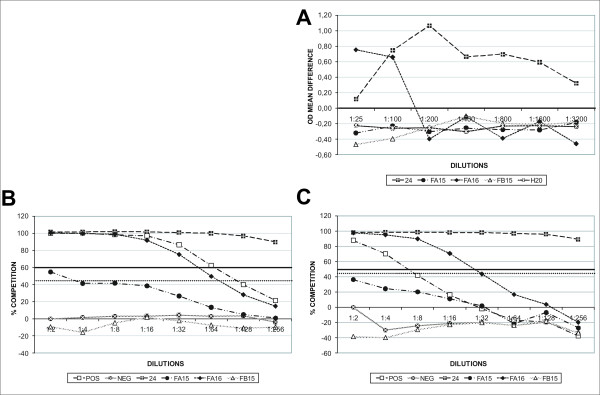
**Serial dilution curves of a panel of reindeer serum samples tested in three commercial bovine ELISA kits for detection of alphaherpesvirus antibodies**. Four samples were selected to illustrate different situations: Serum samples 24 and FA16 were seropositive in all kits; serum sample FA15 was found to be doubtful in Kit B and seronegative in Kits A and C; serum sample FB15 was classified negative in all kits. The positive and negative cattle sera controls from Kits B and C were also titrated. In Kit A there were no controls, but water was tested as a negative control. 3A, 3B and 3C displays Kits A, B and C serial dilutions respectively. In Figure 3A a continuous bold line (—) indicates the cut-off value for Kit A (0.00). In Figures 3B and 3C a continuous bold line (—) indicates the upper cut-off value for the kits (60% for Kit B and 50% for Kit C) while a dotted bold line (···) indicates the lower cut-off values (45% for both kits).

Serial dilution curves are displayed in Figure 3. The curves for Kit A (Figure 3A) displayed some inconstant results for the first dilutions. The curves for Kits B and C (Figure 3B and 3C) were comparable to each other. Serum sample 24 (positive in all kits) was classified as positive in all dilutions while the serum sample FA16 (also classified positive for all kits) became negative at dilution 1:32, 1:64 and 1:200 for Kits C, B and A, respectively. Positive control samples (Kits B and C) became negative at a dilution of 1:128 in Kit B and 1:8 in Kit C.

### Virus neutralization results

The VNT confirmed the ELISA results. All samples that were classified negative by all the three ELISA kits failed to neutralize any of the viruses. All samples classified positive in all kits neutralized CvHV-2, and some of them also neutralized BoHV-1 though at a lower titre. Neutralizing titres ranged for CvHV-2 from 1:2 to 1:256 and for BoHV-1 from 0 to 1:16. No reindeer serum sample neutralized BoHV-1 to a higher titre than CvHV-2 and the biggest difference observed between a sample neutralization of CvHV-2 versus BoHV-1 was of 5 dilutions steps. Samples that were classified as doubtful in the ELISA kits were subsequently retested and classified as negative, and when tested in the VNT they also failed to neutralize any of the viruses. Only one weak positive sample in Kit B, which was doubtful in Kit C and negative in A failed to neutralize CvHV-2, while one sample classified as negative in Kit A but as positive in the other two kits had a low titre for CvHV-2 (1:2). The reindeer positive control neutralized CvHV-2 up to 1:128 and BoHV-1 up to 1:16 while the bovine positive control neutralized BoHV-1 up to 1:32 and CvHV-2 only at 1:2. The coefficient of antigenic similarity between CvHV-2 and BoHV-1 was of R = 8.8. Results are summarized in Table 3.

**Table 3:** Virus neutralization test (VNT) on reindeer sera tested in this study.

**ELISA Kits results**			**No. of samples**		**Neutralizing antibody titre** **[min and max titre] average**	**Difference between CvHV-2 and BoHV-1 in dilution steps**
**A**	**B**	**C**		**CvHV-2**	**BoHV-1**	**[min – max] average**

+	+	+	62	[1:2–1:256] 1:45	[0–16] 1:3	[1-5] 3.2
-	±	-	3	[0]	[0]	0
-	+	±	1	[0]	[0]	0
-	+	+	1	[1:2]	[0]	1
-	-	-	87	[0]	[0]	0

+	+	+	Bovine + control	[1:2]	[1:32]	4
+	+	+	Reindeer + control	[1:128]	[1:16]	3

Neutralizing titres are expressed as the reciprocal of the highest serum dilution that completely prevented a cytopathic effect (CPE). For each virus neutralization, the maximum and minimum titres obtained in each group of samples are presented. Samples were grouped according to the results they obtained in the different ELISA kits (+ for positive; ± for doubtful; – for negative). [0] represents the absence of neutralization. The difference between the neutralizing titres against CvHV-2 and BoHV-1 is presented in dilution steps differences.

## Discussion

Serological results obtained with the three different kits showed that the blocking design kits performed better than the indirect ones as had been concluded for the use of similar kits for BoHV-1 [14], and identified that an alphaherpesvirus serologically related to BoHV-1 is present in semi-domesticated reindeer in Finnmark. The blocking kits were found to work efficiently without any changes to the manufacturers' protocols or pre-defined cut-off values unlike Kit A which could not be used without adaptations.

Data obtained in the virus neutralization strongly indicates that CvHV-2 is most likely the virus present in this reindeer population.

The percentage of seropositive reindeer ranged from 40–42% between kits. The low variation between the kits verified the consistency of the results. In this study, reindeer samples were tested in serological kits designed for bovine sera and it was therefore necessary to verify if the pre-established cut off values could be used for reindeer sera. From the data obtained in the kits with blocking design (B and C) even considerable changes in the cut off values (10% up or downwards) would not significantly change the results.

The Spearman coefficient is based on the ranks, reducing sensitivity to outliers that could affect the Pearson correlation coefficient. The value of Spearman's ρ calculated for each sub-populations of positive or negative results, showed that there was an association between ranks when the two blocking kits were compared as could have been expected given they were based on the same blocking ELISA design. Samples tended to score similar percentages of competition for Kits B and C even when we analyzed only those samples flanking the cut-off lines. The clustering in two populations above and below the cut-off line with similar quantitative and qualitative results was shown to be concurrent with the VNT results with the exception of two samples.

Despite using a different method, Kit A showed qualitative results (animal classified as positive or negative) very similar to the other two kits. Some association within positive results between Kits A and B further showed that the tested ELISA kits correctly classified samples even when using different methods.

Regarding the samples that scored negative in Kit A while positive or doubtful in Kits B and C, one could also consider that the difference may be due to a non specific inhibitory character in the sera or a possible difference in available epitopes for reaction between the two ELISA methods.

The analysis of variability serves as an important tool to study repeatability, and the differences between samples tested in duplicate in the same plate is a good evaluator. A mean variability in OD of 0.06 (5–95% [0.001; 0.123]) for Kit C and of 0.03 (5–95% [0.001; 0.160]) for Kit B are good evidences that gB blocking kits had a better repeatability compared to the indirect ELISA (Kit A), which had a mean variability in OD of 0.37 (5–95% [0.018; 1.188]). It is however important to remember that a direct comparison is difficult since the protocol of Kit A had to be adapted to test reindeer sera. In Kits B and C variability was obtained from the absolute difference between the observed OD for a given sample (|OD_S1 _- OD_S2_|), where S1 and S2 represent the duplicates of a given test sample. In Kit A however, there was an intermediate step for the calculation of the same value (|(OD_IBR1 _- OD_CONTROL1_) – (OD_IBR2 _- OD_CONTROL2_)|), where _CONTROL _represents the control wells, _IBR_the well containing the antigen and 1 and 2 the duplicates. This additional step in Kit A might also have contributed to the higher variability in Kit A versus Kits B and C.

If we consider that analytical sensitivity is the largest dilution of a high-level positive serum in which antibody is no longer detected, we observed a similar pattern for all kits, in which sample 24 remained positive at 1:256 for Kits B and C and at 1:3200 for Kit A. Sample FA16, which was another strong positive (though not as strong as number 24), became negative at 1:200, 1:64, 1:32 for Kits A, B and C respectively.

The abnormal curve observed in Kit A (Figure 3A) was repeated and confirmed and could possibly be explained by unspecific factors in the sera which interfered with the binding of the antibodies.

Given the reduced number of samples tested it is difficult to present a final conclusion for sensitivity, but we might conclude for Kits B and C that they have a good sensitivity as positive samples are still detectable 3 to 4 dilution steps below their testing dilution. Further, it is possible to conclude from the three serial dilution curves, that the blocking design kits presented a more stable curve with a moderate decrease in competition percentage when compared to the indirect ELISA kit where OD values changed abruptly and oscillated even though sensitivity also seemed to be high considering how the positive samples scored.

When comparing the ELISA designs used in this study, it is demonstrated from the serology but also from the variance and serial dilution analysis that the gB blocking design kits should be preferred to the indirect ELISA kit. This was also the situation when testing cattle, where the BoHV-1 gB kits was found more suitable as compared to kits with an indirect ELISA design [14,24-26]. The lower performance of Kit A in this trial may have derived from the adaptations introduced and the conclusions drawn are therefore only valid regarding its adaptation to test reindeer sera as required by the aim of this study.

When comparing the two blocking ELISA kits little differences can be found, though Kit C gave less doubtful results and a slightly better repeatability. The positive control serum of Kit C performed however worse in the dilution analysis compared to the positive control of Kit B, becoming negative at dilutions of 1:8 and 1:128 respectively.

Regarding the VNT, Kramps et al. [14] clarified that VNT did not present sufficient advantages to be the method of choice for cattle. They showed that the ELISA kits had a higher sensitivity and specificity and that they were time and cost saving when large numbers of samples were to be tested.

Even though the ELISA kits compared in this study were designed for cattle, the genetic similarity between BoHV-1 and CvHV-2 was sufficient for all kits to detect reindeer antibodies against CvHV-2. The VNT confirmed this by showing an unequivocal higher neutralization against CvHV-2 with an average difference of three dilution steps to BoHV-1. Neutralization against other alphaherpesviruses was not performed in this study given their unlikely presence in Norway.

The VNT further showed that the cut-off values of the ELISA kits were placed at a correct percentage of competition for Kits B and C and correct OD value for Kit A. Samples classified as doubtful (Kits B and C) where negative in the VNT and only one low positive in Kit B (doubtful in Kit C) and one negative sample in Kit A might have been misclassified by the ELISA kits, if one wishes to consider the VNT as a potential gold standard test for this type of wildlife screening.

The present coefficient of antigenic similarity of 8.8 is in line with previous calculations by Lyaku et al. and Rimstad et al. who calculated it to be 9 and 8.8 respectively, even though the titres against CvHV-2 were lower in this study (1:256 maximum) than in previous ones, where reindeer sera neutralized CvHV-2 up to 1: 1024 and 1:512 respectively [18,20].

It is important to clarify that the VNT was used mostly to confirm the presence of another alphaherpesvirus than BoHV-1, as would be expected given the BoHV-1 free status in cattle in Norway, and not specifically to compare the performance of ELISA versus VNT despite the agreement found between the two types of tests.

Kits B and C used as antigen the gB glycoprotein which is strongly immunogenic and induces a humoral response that appears in an early stage of infection [27]. This response persists two to three years after infection in cattle [28]. Because of the persistence of anti-gB antibodies, as well as the fact that the gB antigen is genetically conserved between alphaherpesviruses of ruminants, gB can be regarded as an ideal antigen for serology in wild animals for which the time of infection is unknown and no validated serological tests are commercially available.

## Conclusion

The blocking ELISA kits using gB as antigen were found to be preferable to use in serosurveys for alphaherpesvirus in reindeer. Furthermore the choice of a blocking ELISA enables all ELISA components to be used and thus gives both economical and time saving advantages.

With 40% of tested animals presenting antibodies against alphaherpesviruses, our results indicate that an alphaherpesvirus infection is present in reindeer in Finnmark County.

The virus neutralization results, associated to the inexistence of BoHV-1 in Norway, strengthened and confirmed the hypothesis that the virus present in this population is indeed CvHV-2 and that a blocking ELISA commercial kit can efficiently be used to screen reindeer for the presence of antibodies against this virus.

These results, in combination with the knowledge of the biological and economical importance of the closely related BoHV-1 infection in cattle, should encourage further studies of the distribution and impacts of CvHV-2 infection in reindeer in Scandinavia.

## Competing interests

The authors declare that they have no competing interests.

## Authors' contributions

CDN and MT designed the experiment and analyzed the data. CDN, MT and MR performed the experiment. CDN and NGY performed the statistical analysis. ER and CDN performed the virus neutralization assay. CDN, ER and MT, drafted the manuscript. MR and NGY further helped to draft the manuscript. All authors read and approved the final manuscript.
